# Effects of preconditioning of electro-acupuncture on postoperative cognitive dysfunction in elderly: A prospective, randomized, controlled trial

**DOI:** 10.1097/MD.0000000000007375

**Published:** 2017-06-30

**Authors:** Qi Zhang, Ya-Nan Li, Yang-Yang Guo, Chun-Ping Yin, Fang Gao, Xi Xin, Shu-Ping Huo, Xiu-Li Wang, Qiu-Jun Wang

**Affiliations:** Department of Anesthesiology, the Third Hospital of Hebei Medical University, No.139, Ziqiang Road, Shijiazhuang City, Hebei, China.

**Keywords:** aged, electro-acupuncture, postoperative cognitive dysfunction

## Abstract

Electro-acupuncture is a burgeoning treatment using the needle inserting into the body acupoints and the low-frequency pulse current being electrified by an electric acupuncture machine. This study was designed to evaluate the effects of preconditioning of electro-acupuncture on postoperative cognitive dysfunction in elderly.

Ninety patients scheduled spine surgery were randomly assigned into 2 groups using a random number table: control group (group C) and electro-acupuncture group (group EA). In group EA, electro-acupuncture was applied on Baihui, Dazhui, and Zusanli acupoints 30 minutes before anesthesia. At 0 minute before treatment of electro-acupuncture, 1 hour after skin incision and surgery completed (T_1–3_), blood samples were taken for detection of interleukin (IL)-6, IL-10, and S100β by enzyme-linked immunosorbent assay. The total dose of remifentanil and propofol during surgery were recorded. Mini-Mental State Examination was applied to evaluate the cognitive function of patients at 1 day before surgery and 7th and 30th day after surgery.

The results showed that compared with group C, score of MMSE increased after surgery, the serum concentration of IL-6, IL-10, and S100β decreased at 1 hour after skin incision, and surgery completed in group EA. Moreover, the total dose of remifentanil and propofol reduced during surgery in group EA.

The present study suggests that preconditioning of electro-acupuncture could improve the postoperative cognitive function, and the reduction of inflammatory reaction and brain injury may be involved in the mechanism.

## 1 Introduction

Postoperative cognitive dysfunction (POCD), a common complication of central nervous system (CNS) with the characteristics of insanity, anxiety, personality changes, memory impairing, and so on, is a major clinical issue in geriatric surgical patients after anesthesia in the elderly (≥65 years).^[1–2]^ Its incidence varies from 20% to 79% in cardiac surgery and 4.1% to 22.3% in noncardiac surgery.^[3–4]^ POCD brings physical, psychological, and economic burden to patients and their families. Gonda et al^[5]^ found that persistent cognitive dysfunction is also important clinically, because it decreases coping capacities and influences therapeutic compliance and cooperation as well. Therefore, it is urgent to find effective interventions for POCD.

Acupuncture is a kind of special treatment for systemic diseases through the conduction of meridians and acupoints in China for over 2000 years.^[6]^ Electro-acupuncture (EA) is new therapeutic tool decreasing brain injury based on acupuncture.^[7]^ Combined with the effect of electrical stimulation, it can produce different treatment effect at different acupoints by adjusting the waveform, time, frequency, and intensity parameters.^[8–10]^ Studies showed that treatment with EA could improve cognitive function in patients with Alzheimer disease.^[11]^

Our previous study suggested that preconditioning of EA could reduce the incidence the postoperative cognitive dysfunction in aged rats. However, whether it could improve the cognitive function of elderly patients is still confused. The purpose of this study is to evaluate the effects of preconditioning of electro-acupuncture on postoperative cognitive dysfunction in elderly, and the results could provide a new direction for the prevention of POCD in elderly.

## 2 Materials and methods

### 2.1 Ethics statement

The present study has been performed with the approval of the ethics committee of the Third Hospital of Hebei Medical University and is in compliance with the Helsinki Declaration. The informed consents of the study were collected from all the candidate subjects. All patients have signed informed consent and study was conducted in accordance with the approved guidelines and informed consent from each subject

### 2.2 Patients

**Figure 1:**
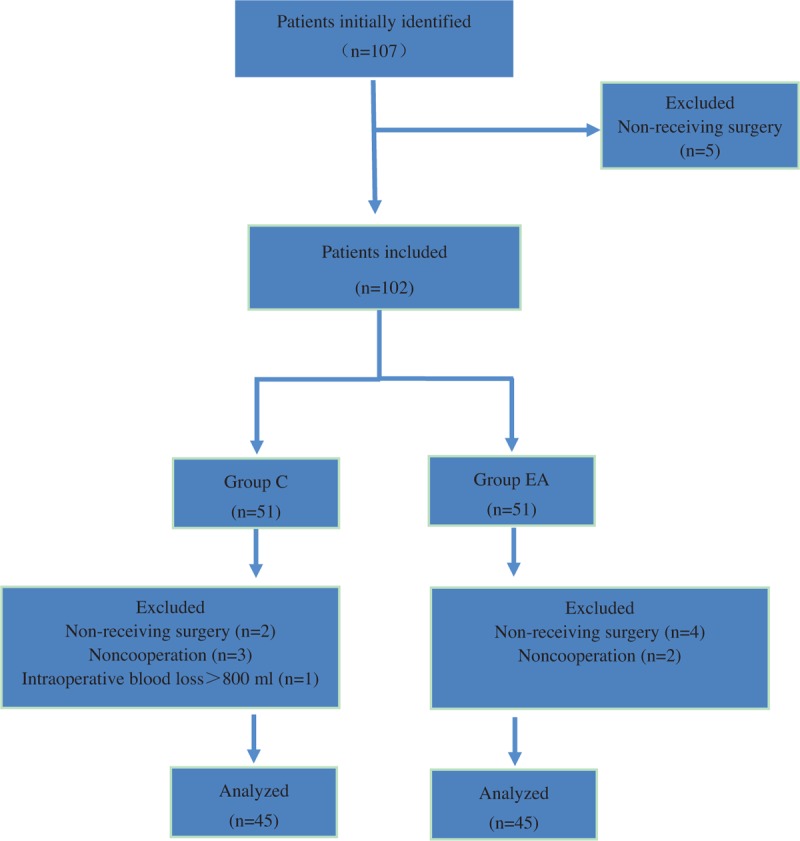
Experimental flow of this study.

From October 2016 to April 2017, 107 aged patients (>65 years old) undergoing spine surgery in the Third Hospital of Hebei Medical University were enrolled. As shown in Figure 1, because of noncooperation, excessive intraoperative blood loss and nonreceiving surgery, 17 patients were lost. The sample size of the study was calculated according to previous studies and was based on a pilot study. Inclusion criteria of the present study were as follows: American Society of Anesthesiologists’ physical status classes I and II; no history of traumatic brain injury, neurological diseases, and alcohol abuse; no cognitive dysfunction; no severe vision or hearing impairment and no obvious abnormality of heart, lung, liver, and kidney function. If the operation time is greater than 3 hours, the intraoperative blood loss is more than 800 mL, or the patients did not undergo surgery, they will be excluded from this study. The characteristics of participants were shown in Table 1. Then patients were randomly assigned into 2 groups with random number table: control group (group C) and electro-acupuncture group (group EA).

**Table 1:**
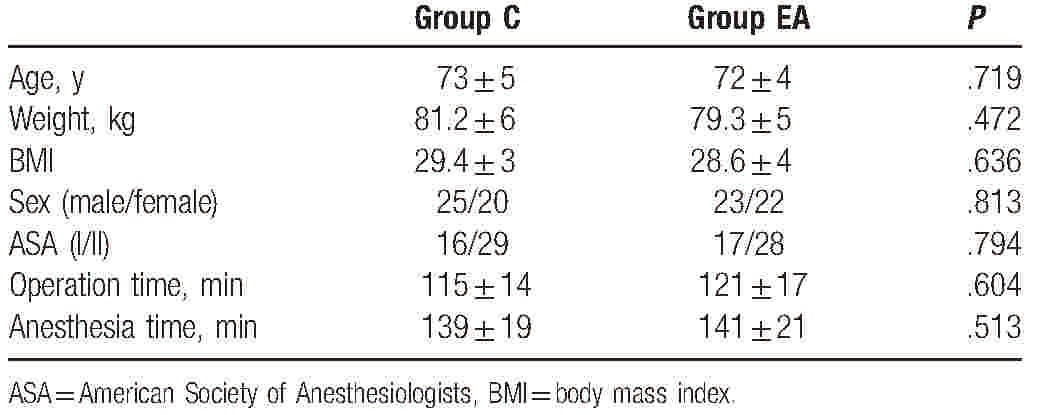
General information of patients (n = 45).

### 2.3 Intervention methods

After entering the operating room, electrocardiogram (ECG), pulse oxygen saturation (SpO_2_), respiratory rate (R), PetCO_2_, bispectral index (BIS), and invasive blood pressure of patients were recorded. The patients of group EA received EA treatment at Baihui (GV20), Dazhui (GV14), and Zusanli (DT36) according to the textbook of experimental acupuncture by EA stimulator instrument (Model G6805; SMIF, Shanghai, China) 30 minutes before anesthesia. Baihui is located at the intersection of the sagittal midline and the line linking the 2 ears and Dazhui is located between the seventh cervical vertebra and the first thoracic vertebra. Zusanli is located outside the calf, 3 inches below the Dubi acupoint. The following parameters were applied for the treatment: sparse-dense wave (2/15 Hz) and intensity at 1 mA. EA treatment continued until the end of surgery.

The method of anesthesia was standardized for 2 groups. The patients were induced using 0.1 to 2 μg/kg sulfentanil, 0.05 to 0.2 mg/kg midazolam, 0.3 mg/kg etomidate, and 0.6 mg/kg cisatracurium. A reinforced catheter was inserted after 2 minutes of cisatracurium administration. Ventilation frequency was set to 12 times per minute, inspiratory expiratory ratio to 1.0:1.5, inhaled oxygen concentration to 100%, oxygen flow to 2 L/min, and PaCO_2_ was maintained within the physiologic limits (35–45 mm Hg). The entire course of anesthesia was maintained using propofol 4 to 6 mg/(kgh) and remifentanil 0.1 to 0.3 μg/(kgmin), which sustained the changing of BP and HR within 20% of the initial levels and the BIS value range from 40 to 60. EA treatment, remifentanil, and propofol were stopped when the skin was cut off.

### 2.4 Data collection and index detection

At 0 minute before treatment of EA, 1 hour after skin incision and surgery completed (T_1–3_), 4 mL blood samples were taken for detection of interleukin (IL)-6, IL-10, and S100β by ELISA, and the dosage of remifentanil and propofol during surgery was recorded.

At 1 day before surgery and 7th and 30th day after surgery, cognitive function of patients was tested using Mini-Mental State Examination (MMSE) by a well-trained investigator who was blind to the study grouping. MMSE is a scale of cognitive function which could evaluate patients’ time-directed force, site-directed force, immediate memory, attention and computing power, delayed memory, language, and visual space. It contains 30 questions, and patients will obtain 1 point if answer is correct, but 0 point if the answer is wrong. The total scores less than 27 indicate cognitive dysfunction.^[12]^

### 2.5 Statistical analysis

All data were analyzed by SPSS (version 21.0 for Windows, SPSS Inc, Chicago, IL). Measurement data of normal distribution were reported as the mean ± SD. Comparisons between 2 groups were performed with *t* test; comparisons among different groups were done with 1-way analysis of variance (ANOVA). Two-tailed probability value of *P* < .05 was considered as statistically significant.

## 3 Results

### 3.1 Demographic data and anesthetic dosage

**Figure 2:**
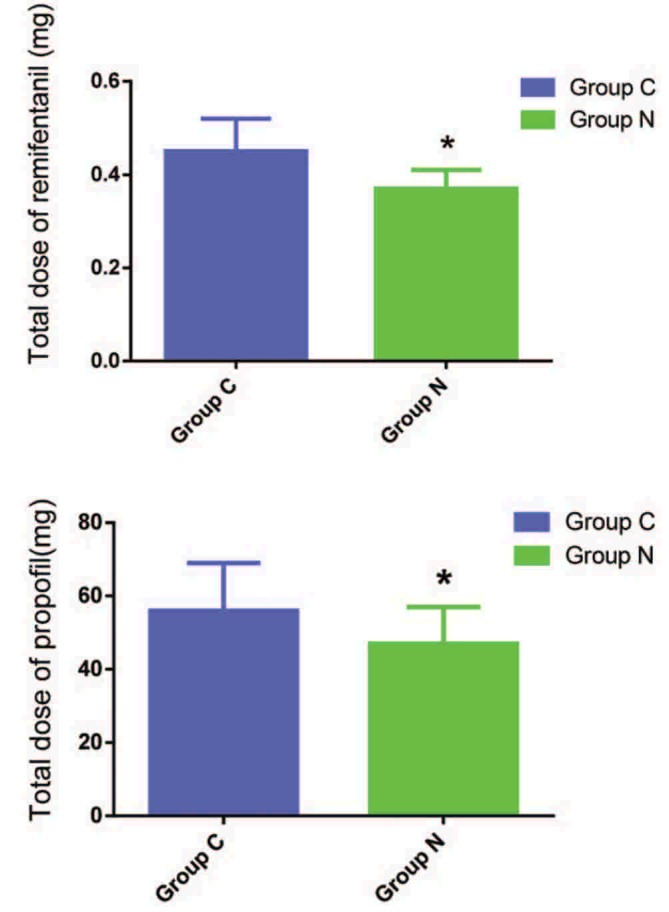
Total dose of remifentanil and propofol in 2 groups. Compared with group C, ^∗^*P* < .05.

In all, 107 patients were enrolled, and assigned to 2 groups (11 patients were lost because of nonreceiving, 5 patients were lost because of noncooperation, and 1 patient was lost because of intraoperative blood loss >800 mL). As shown in Table 1, there were no differences in age, weight, BMI, sex, ASA, operation time, and anesthesia time. Compared with group C, the dose of remifentanil (*t* = 2.376, *P* = .031) and propofol (*t* = 2.687, *P* = .009) during surgery decreased in group EA (Fig. 2).

### 3.2 Scores of MMSE

**Figure 3:**
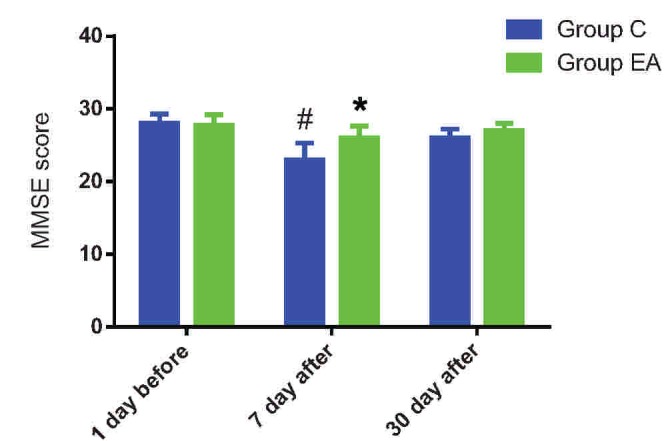
Score of MMSE at different time points in 2 groups. Compared with group C, ^∗^*P* < .05. Compared with the value before surgery, ^†^*P* < .05. MMSE = Mini-Mental State Examination.

Before the surgery, there was no difference in MMSE score between group C and group EA. As shown in Figure 3, after the surgery, the MMSE score in group EA remained unchanged at 7th and 30th day after surgery. For patients in group C, however, the MMSE score decreased significantly at 7th day after surgery (*t* = 2.539, *P* = .023) when compared with its own preoperative value, and also decreased significantly (*t* = 2.677, *P* = .01) when compared with the patients in group EA.

### 3.3 The level of IL-6, IL-10, and S100β

**Figure 4:**
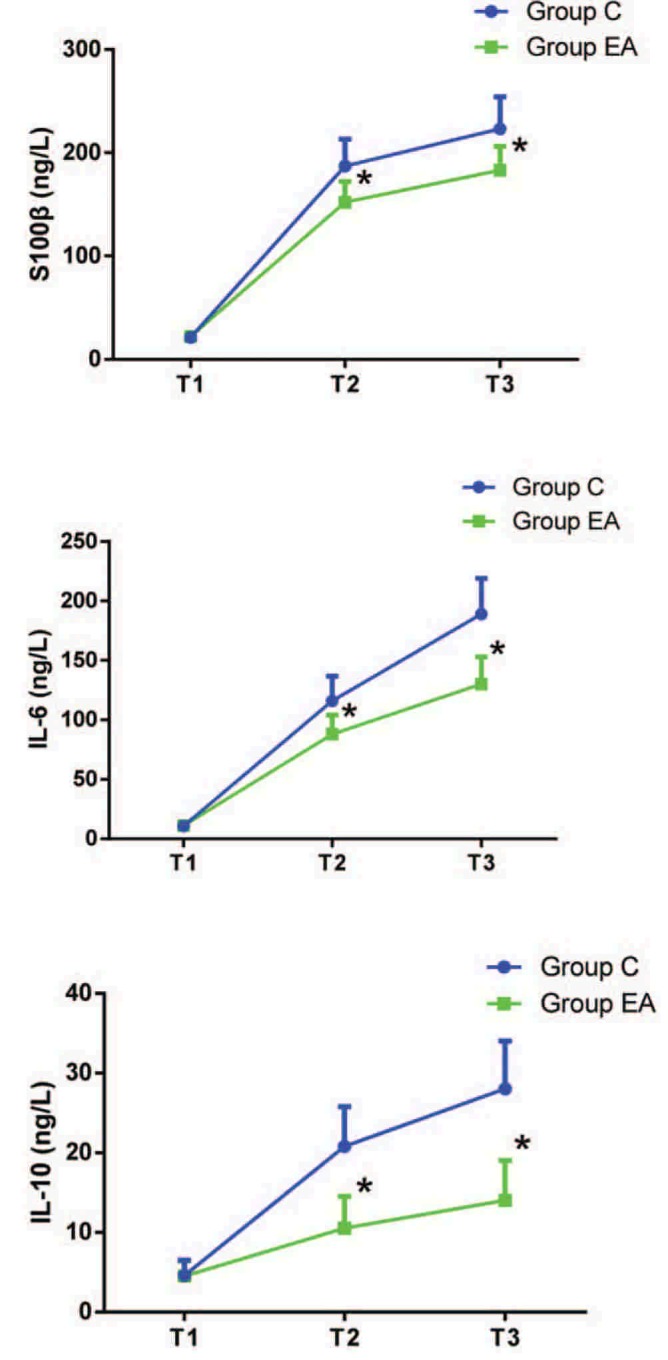
The level of IL-6, IL-10, and S100β at different time points. T_1_ = 0 minute before treatment of electro-acupuncture, T_2_ = 1 hour after skin incision, T_3_ = surgery completed. Compared with group C, ^∗^*P* < .05.

There was no significant difference in IL-6 (*t* = 0.864, *P* = .393), IL-10 (*t* = 1.316, *P* = .187), and S100β (*t* = 1.281, *P* = .207) between 2 groups at T_1_. As shown in Figure 4, compared with group C, the level of IL-6 (T_2_: *t* = 2.109, *P* = .043; T_3_: *t* = 2.423, *P* = .016), IL-10 (T_2_: *t* = 2.283, *P* = .037; T_3_: *t* = 2.374, *P* = .026), and S100β (T_2_: *t* = 2.409, *P* = .021; T_3_: *t* = 2.475, *P* = .013) reduced at T_2_ and T_3_ in group EA.

## 4 Discussion

In the present study, we found that preconditioning of EA could improve the cognitive function of aged patients receiving spine surgery and reduce the total dose of anesthetic. The reduction of inflammatory response and brain injury may be involved in the mechanism. Our previous study and some scholars proposed pretreatment with EA was able to attenuate the POCD in aged rats.^[13]^ However, whether the preconditioning of EA could improve the postoperative cognition in elderly is still not clear. This study was undertaken to investigate the effects of preconditioning of EA on postoperative cognitive dysfunction in elderly.

Postoperative cognitive dysfunction is 1 of the common complications in elderly receiving spine surgery. Because of major trauma, long operation and anesthesia time, and excessive intraoperative blood loss, the patients receiving spine surgery easily suffer POCD.^[14]^ Although the pathogenesis of POCD remains to be clarified, many studies have identified that hippocampal neuronal apoptosis^[15]^ and neuroinflammation^[16]^ are major risk factors to POCD. Due to degenerative changes of the structure and function of the tissues and organs, it cannot provide maximum additional safety requirements for disease, surgery, trauma, and rehabilitation. Moreover, comparing with lots of disease before surgery, it is easy for elderly patients to develop various complications after operation. A lot of studies have been conducted to investigate that elderly patients are more likely to suffer from POCD after surgery than younger patients.^[17]^

Acupuncture has an effect on the nervous system and immune system, which has analgesic effect, immune regulation, and regulation on the function of organs.^[18]^ Chinese traditional medicine proposes that Dazhui and Baihui belong to Du meridian points, which closely related to brain function. There is evidence showing that preconditioning of EA at Baihui and Dazhui acupoints can alleviate cerebral ischemia reperfusion injury induced cognitive impairment in elderly.^[19]^ Zusanli is an important acupoint of health care. Therefore, we chose these 3 acupoints for electrical stimulation.

As mentioned above, neuroinflammation is closely related to the pathogenesis of POCD.^[16]^ IL-6 is mainly produced by mononuclear macrophages, which is the key proinflammatory factor in the acute stage of trauma. IL-10, an important anti-inflammatory cytokine in vivo, is mainly produced by activated T, B, and mononuclear cells. The changes of IL-6 can reflect the degree of surgical stimulation and injury,^[20]^ whereas IL-10 can inhibit the release of inflammatory cytokines IL-6 and tumor necrosis factor (TNF)-α, which play an anti-inflammatory role.^[21]^ As a marker protein of glial cells, S100β protein is mainly expressed in astrocytes and Schwann cells.^[22]^ High concentration of S100β protein is toxic to neurons, and is closely related to POCD. Some studies suggested that serum S100β protein content can be used as an important indicator to evaluate the incidence, course, and incidence of POCD. In this study, our data indicated that preconditioning of EA could decrease the level of IL-6, IL-10, and S100β protein, inhibited inflammatory response, and brain injury in elderly.

There is no denying that the present study has several limitations. First, the number of patients is relatively small. Second, we only studied elderly patients undergoing spine surgery. So the effects of preconditioning of EA on postoperative cognitive dysfunction in patients of other age groups or receiving other surgeries need further study.

In conclusion, preconditioning of EA could improve the postoperative cognitive function and the reduction of inflammatory reaction, and brain injury may be involved in the mechanism.
